# The Hydrogen Sulfide-Vitamin B12-Folic Acid Axis: An Intriguing Issue in Chronic Kidney Disease. A Comment on Toohey JI: “Possible Involvement of Hydrosulfide in B12-Dependent Methyl Group Transfer”. *Molecules* 2017, 22, 582, pii: E582

**DOI:** 10.3390/molecules22071216

**Published:** 2017-07-19

**Authors:** Giuseppe Cianciolo, Maria Cappuccilli, Gaetano La Manna

**Affiliations:** Department of Experimental, Diagnostic and Specialty Medicine (DIMES)—Nephrology, Dialysis and Transplantation Unit, St. Orsola Hospital, University of Bologna, Via G. Massarenti 9 (Pad. 15), 40138 Bologna, Italy; giuseppe.cianciolo@aosp.bo.it (G.C.); maria.cappuccilli@unibo.it (M.C.)

**Keywords:** cardiovascular disease, chronic kidney disease, hydrogen sulfide, methylation, sulfane sulfur, S-adenosylmethionine methyl transferase

Dear Editor, 

We read with great interest the recent article by John I. Toohey entitled “Possible Involvement of Hydrosulfide in B12-Dependent Methyl Group Transfer”, recently published in Molecules 2017, and we wish to discuss some additional insights raised by this important issue into the nephrological area [1].

Sulfur in the form of sulfane sulfur or hydrogen sulfide (H_2_S) has been shown to have numerous regulatory effects in several biological systems. The author addresses the hypothesis that the sulfur atom could also be involved in vitamin B12-dependent methyl group transfer. The impairment of this metabolic pathway leads to decreased methionine synthesis, decreased S-adenosylmethionine (SAM) availability, and ultimately to hypomethylation of essential sites. The methylation of molecules such as creatine, DNA, RNA, phosphatidylcholine, and many neurotransmitters occurs through a transmethylation process. The final donor of methyl groups in this pathway is usually SAM, and the end-product of methyl group transfer from SAM is homocysteine which is remethylated by B12-dependent methyltetrahydrofolate (CH_3_-THF)-homocysteine S-methyltransferase, also called methionine synthase (MS). SAM-mediated methylation reactions are indirectly dependent on the B12-dependent methylation of homocysteine [2]. During the metabolism of homocysteine to cysteine by the enzymes cystathionine beta-synthase (CBS) and cystathionine gamma-lyase (CSE), H_2_S is also produced as a side product. H_2_S is an angiogenic agent with antioxidant and vasorelaxing properties.

Hyperhomocysteinemia causes downregulation of CBS and CSE, resulting in H_2_S depletion, which in turn leads to vascular damage, vascular disease, and subsequently to deterioration of endothelial function [3].

At each stage of chronic kidney disease (CKD), the risk of cardiovascular mortality is increased several-fold compared to that of the general population, particularly when CKD progresses to end-stage renal disease (ESRD) [4]. Various possible underlying pathophysiological pathways have been proposed to account for this excess mortality. Hyperhomocysteinemia occurs in about 85% of CKD patients because of altered renal metabolism and impaired excretion [5]. Homocysteine is currently regarded as an independent predictor of cardiovascular morbidity and mortality in ESRD patients [6,7].

Folic acid and vitamins B12 and B6 are important regulators of homocysteine metabolism. Vascular cells—in particular vascular endothelial cells—are believed to be particularly susceptible to elevated levels of homocysteine. This cell type does not express CBS (the first enzyme of the hepatic reverse transsulfuration pathway) nor betaine homocysteine methyl transferase (BHMT), which catalyzes the alternate remethylation pathway in the liver using betaine as a substrate. Therefore, endothelial cells can eliminate homocysteine only by the folic acid- and vitamin B12-dependent remethylation pathway regulated by methylenetetrahydrofolate reductase (MTHFR) and MS. For this reason, a normal activity of both MTHFR and MS is essential to prevent homocysteine increase to a pathological level in vascular endothelial cells.

In addition, the high prevalence of hyperhomocysteinemia in patients with CKD has sparked interest in the potential role of hyperhomocysteinemia as a risk factor for the progression of CKD [8,9]. Hyperhomocysteinemia is known to promote atherogenesis and atherothrombosis through different mechanisms: first, the endothelial damage due to reactive oxygen species generated by homocysteine metabolism; secondly, the inhibition of nitric oxide synthase activity by homocysteine resulting in endothelial malfunction. Additional proatherogenic triggers are represented by dysregulated methylation of proteins and DNA resulting in abnormal vascular smooth muscle cell proliferation and increased lipid peroxidation [10].

However, previous trials based on supplementation with folic acid and B vitamins (including cyanocobalamin) did not show unequivocal results in terms of cardiovascular and renal outcomes [8,11,12]. Actually, in the general population as well as in CKD patients, whether the benefits of folic acid therapy are due to its direct effect or to a reduction of hyperhomocysteinemia still remains an open question. H_2_S is the third endogenous gaseous mediator after nitric oxide (NO) and carbon monoxide [13], and has been implicated in several physiological processes—namely vascular smooth muscle relaxation, inhibition of vascular smooth muscle cell proliferation, and blood pressure lowering [14]. H_2_S metabolism changes may contribute to the development of uremia-accelerated atherosclerosis in CKD patients with diabetic nephropathy [15]. 

Nevertheless, the cardiovascular protective mechanism of H_2_S in CKD patients is still unclear. Recently, Feng et al. showed low plasma levels of H_2_S to be associated with increased cardiovascular risk and mortality in CKD patients, highlighting a pathological link feasibly mediated by the cPKCβII/Akt pathway and VCAM-1/ICAM-1 upregulation [16].

Protein kinase CβII (PKCβII) is a member of the conventional subfamily of protein kinase C (PKC) isozymes, and its activation has emerged as a major contributor to the pathogenesis of atherosclerosis [17] and heart failure [18]. In this context, the article by John Toohey that highlights the links among reduced availability of H_2_S, reduced methionine synthesis, low SAM, and hyperhomocysteinemia adds a further piece to the puzzle of CVD and CKD progression.

An unsolved issue is represented by question of whether the low H_2_S plasma levels observed in CKD/ESRD patients result from downregulation of CBS and CSE mediated by hyperhomocysteinemia or if this decrease should be attributed to other causes.
